# Draft Genome Sequence of Marine Cyanobacterium *Synechococcus* sp. Strain NKBG15041c

**DOI:** 10.1128/genomeA.00954-13

**Published:** 2013-11-27

**Authors:** Tomoko Yoshino, Toru Honda, Masayoshi Tanaka, Tsuyoshi Tanaka

**Affiliations:** Division of Biotechnology and Life Science, Institute of Engineering, Tokyo University of Agriculture and Technology, Tokyo, Japana; JST, CREST, Tokyo, Japanb

## Abstract

*Synechococcus* sp. strain NKBG15041c was isolated as a fast-growing marine cyanobacterium. Genetic transformation techniques using this strain have been well established for metabolic engineering. Here we report the draft genome sequence for this strain, consisting of 44 contigs containing a total of 3,180,043 bp and 3,224 putative protein-coding genes.

## GENOME ANNOUNCEMENT

Cyanobacteria are potentially employed for sustainable bioenergy generation because of their superior photosynthesis capacity for converting carbon dioxide into carbohydrates (1, 2) without competition with food or feed production. Furthermore, cyanobacteria are more amenable to genetic manipulation for installing pathways or enhancing their productivity (3, 4). Recently, cyanobacteria have been used for the production of valuable compounds, including alkane (5), butanol (6, 7), acetone (8), and isoprene (9), through the expression of heterogeneous genes using *Synechocystis* sp. PCC6803 and *Synechococcus elongates* PCC7942. Thus, these species are promising hosts for the production of useful compounds by metabolic engineering. They are naturally competent and are known to transport DNA across the cell membrane. These transformable unicellular strains are commonly used for genetic engineering because their transformation efficiencies are much higher than those of other strains.

*Synechococcus* sp. strain NKBG15041c was isolated as a fast-growing marine cyanobacterium from coastal seawater at Okinawa prefecture in Japan (10). Strain NKBG15041c is not naturally competent, and the gene transfer technique of transconjugation was first established in marine cyanobacteria using this strain (11). Several biotechnological applications of this strain have been demonstrated by genetic engineering (12, 13), and this strain has demonstrated the potential to provide host cells for biofuel production. Here, we report the draft genome sequence of the marine cyanobacterium *Synechococcus* sp. strain NKBG15041c.

*Synechococcus* sp. NKBG15041c was grown in marine BG11 medium (ATCC catalogue, medium no. 617) under continuous illumination at 26° C for 1 to 2 weeks in a reciprocating shaker. The genomic DNA was extracted and purified using a DNeasy plant minikit (Qiagen). One microgram of genomic DNA was provided for sequencing. The genome sequencing was carried out using a 454 GS Junior platform (Roche), generating 146,925 reads, for a total of 68,209,717 bp, with 19-fold average coverage. With the Newbler assembler version 2.5 software (Roche), the obtained sequences were assembled into 44 contigs (>500 bp) with an *N*_50_ contig size of 168,293 nucleotides and a total length of 3,180,043 bp with a GC content of 49.3%. Genome annotation of the obtained scaffolds was performed using Glimmer 3.02 software and BLAST searches against a nonredundant protein sequence database. The genome of the strain contains 3,224 predicted coding regions, 29 tRNA genes, and 3 rRNA genes. tRNA and rRNA genes were predicted using Genetyx Ver. 10 and the RNAmmer Prediction Server, respectively (14). The 16S rRNA gene was obtained from the annotation and it was 98% identical to that of the closely related species *Synechococcus* sp. PCC 7002. The total open reading frames (ORFs) of strain NKBG15041c (total ORFs, 3,224) were compared with those of *Synechococcus* sp. PCC 7002 (total ORFs, 3,187) by BLASTp. NKBG15041c has 2,220 orthologs (E value <1 E^[minus]30^, >40% identity) with this strain.

### Nucleotide sequence accession numbers.

The *Synechococcus* sp. strain NKBG15041c genome sequence and annotation data have been deposited in DDBJ/EMBL/GenBank under accession no. BAUB00000000. The version described in this paper is version BAUB01000000.
